# Pre-Existing Humoral Immunity Enhances Epicutaneously-Administered Allergen Capture by Skin DC and Their Migration to Local Lymph Nodes

**DOI:** 10.3389/fimmu.2021.609029

**Published:** 2021-03-26

**Authors:** Pierre-Louis Hervé, Camille Plaquet, Noémie Assoun, Nathalie Oreal, Laetitia Gaulme, Audrey Perrin, Adeline Bouzereau, Véronique Dhelft, Jean-Louis Labernardière, Lucie Mondoulet, Hugh A. Sampson

**Affiliations:** ^1^ Research and Innovation, DBV Technologies, Montrouge, France; ^2^ Research and Innovation, DBV Technologies, New York, NY, United States

**Keywords:** skin dendritic cells, epicutaneous delivery, allergen capture, Fc receptors (FcR), preexisting immunity

## Abstract

Due to its richness in antigen presenting cells, e.g., dendritic cells (DC), the skin has been identified as a promising route for immunotherapy and vaccination. Several years ago, a skin delivery system was developed based on epicutaneous patches allowing the administration of antigen through intact skin. Using mouse models, we have shown that epicutaneous allergen application leads to a rapid uptake and transport of allergen-positive cells to skin-draining lymph nodes (LN). This occurred primarily in animals previously sensitized to the same allergen. In that context, we sought to better understand the role of the specific preexisting immunity in allergen capture by skin DC and their subsequent migration to LN. Specifically, we investigated the role of humoral immunity induced by sensitization and the involvement of IgG Fc receptors (FcγR). Epicutaneous patches containing fluorescently-labeled ovalbumin (OVA) were applied to naïve mice that had previously received either sera or purified IgG isolated from OVA-sensitized mice. To investigate the involvement of FcγR, animals received 2.4G2 (anti-FcγRII/RIII) blocking antibody, 24 hours before patch application. Mice that received sera or purified IgG originating from OVA-sensitized mice showed an increase in the quantity of OVA-positive DC in skin and LN. Moreover, the blockade of FcγR reduced the number of OVA-positive DC in LN to a level similar to that observed in naïve animals. Overall, these results demonstrate that preexisting specific-IgG antibodies are involved in allergen capture by skin DC following EPIT through the involvement of antigen-specific IgG-FcγR.

## Introduction

The skin barrier is comprised of a dense network of antigen presenting cells (APC), including dendritic cells (DC), such as Langerhans cells (LC), that reside in the epidermal layer (1). These DC provide immune-surveillance by “sensing” pathogens passing into the stratum corneum and play a central role in activating adaptive immunity. Due to this feature, skin has been clearly identified as a promising route for vaccination and immunotherapy. Several years ago a novel epicutaneous delivery system was designed for epicutaneous immunotherapy (EPIT) for the treatment of food allergy (2). This system utilized a patch (Viaskin™) that forms an occlusive condensation chamber where allergen is solubilized by skin humidity and delivered across the stratum corneum to skin DC (2–6). Previous studies demonstrated that allergens applied on intact skin *via* epicutaneous patches efficiently promote down-modulation of allergen-specific immune response in sensitized animal models in association with the induction of Tregs (2, 5, 6). This distinctive response could be related to the unique targeting of LC, which are mainly oriented to promote tolerance (4, 7, 8). It appeared that this tolerogenic immune-modulation could be obtained only when the antigen was administered on intact, uninflamed skin (9). Previous results also suggested that the preexisting immunological status of patch-treated mice had a significant impact on the antigen uptake by skin DC and their migration to the draining lymph nodes (4). Indeed, the capture of patch-administered ovalbumin (OVA) by skin DC was more efficient and occurred more rapidly in OVA-sensitized mice than in naïve animals. Remarkably, the migration of these OVA-positive DC to lymph nodes was observed only in OVA-sensitized mice. In that context, the aim of the present study was to better understand the impact of preexisting specific immunity on allergen capture by skin DC and their migration to draining lymph nodes, with a focus on humoral immunity. To that end, we first measured the expression of different antibody Fc receptors on the surface of skin DCs. Then, using a passive transfer model, we evaluated the capacity of the specific humoral response induced in sensitized animals to promote allergen capture by skin DC and their migration to draining lymph nodes. Finally, using a blocking antibody, we further investigated the specific role of IgG and IgG Fc receptors (FcγR). Our results showed that the humoral response elicited by OVA sensitization increased the capacity of skin DC to capture epicutaneously-administered OVA, leading to the migration of a higher number of OVA-positive DC to local draining lymph nodes. Moreover, our results demonstrated that IgG is the main class of antibody in mice involved in this effect.

## Materials and Methods

### Animals and Ethics

BALB/c mice were purchased from Charles River (Lyon, France) and housed under conventional conditions (DBV Technologies, Montrouge, France, agreement number #A92-049-02). Experiments have been performed according to the European Community rules of animal care, and with permission of the French government (authorization #13305).

### Sensitization of Mice

Mice were sensitized subcutaneously on days 0 and 7 with 10 mg of OVA grade V (Sigma) and 1.6 mg aluminum hydroxide (Sigma Aldrich) in 200 µl of PBS 1X. Two weeks after the end of sensitization phase, blood samples were collected by submandibular puncture into microtubes containing EDTA (Greiner Bio-One) and centrifuged at 3000 x g for 10 minutes to collect plasma. Plasma samples were then pooled. The quality of the sensitization has been controlled for each individual mouse and for each pool by measuring OVA-specific IgE, IgG1 and IgG2a using a quantitative ELISA as previously described (2).

### Purification of Antibodies and Passive Transfer of Serum

IgG antibodies were purified from pooled sera using Nab Protein G Spin Kit (Thermo Scientific). The flow-through, corresponding to IgG-depleted serum was collected separately. This flow- through contained similar levels of IgE compared to the pooled sera, but no detectable IgG. Purified IgG were dialyzed against PBS 1X and concentrated to an appropriate volume using Vivaspin column (Merck Millipore). Purified IgG or pooled sera were sterile-filtered on a 0.22 µm filter and injected intraperitoneally into recipient mice (500 µL per mice). For each experiment, the quantities of OVA-specific IgG1 and IgE to be injected were determined using a quantitative ELISA. Of note, the injection of 500 µL of pooled sera (the maximum intraperitoneal volume authorized by our ethical guidelines) led to slightly lower titers of specific IgG1 in naïve recipient mice than in sensitized donor mice (see **Figure S1** for representative data). Specific IgE and IgG2a titers remained low or just above the level of detection in recipient mice (data not shown).

### Preparation of OVA-AF488 Patches and Application to Mice

Epicutaneous patches were loaded dropwise with 100 µg of Alexa-Fluor^®^-488 (AF-488) conjugated ovalbumin (Life Technologies). Patches were dried at 30°C for 1 hour in a ventilated oven and stored at 4°C. Before patch application, mice were anaesthetized with ketamine and xylazine (50 and 10 mg/kg, respectively) and hair on the back was removed using electric clippers and depilatory cream (Reckitt Benckiser). Patches containing OVA-AF-488 were applied the following day and secured using an Urgoderm^®^ bandage (Urgo Laboratories). Patches were maintained for 6 or 48 hours based on optimal timepoints previously defined (4).

### Injection of IgG Receptor Blocking Antibodies

Mice received 500 µg of anti-FcγRII/RIII (clone 2.4G2, Bio X Cell) or rat IgG2b as isotype control (clone LTF-2, Bio X Cell) by intraperitoneal injection. To avoid any non-specific anaphylactic reactions, all mice (including isotype control) received 200 µg of the anti-histamine triprolidine hydrochloride (Sigma) by intraperitoneal injection 30 minutes before injecting the monoclonal antibodies. Mice received patches 24 hours later.

### Collection of Brachial Lymph Nodes (BLNs) for Flow Cytometric Analysis

BLNs were harvested in 2 mL of RPMI containing 0.26 U/mL Liberase TL and 25 µg/mL DNase I (Sigma Aldrich). Each BLN was flushed using a syringe and incubated for 20 min at 37°C. The enzymatic reaction was then stopped with 250 µL of EDTA (100 mM). Cells were homogenized with a 100 µm cell strainer in magnetic-activated cell sorting (MACS) buffer (Miltenyi Biotec) and counted.

### Collection of Skin Samples for Flow Cytometric Analysis

A skin sample corresponding to the patch application area was harvested using an 8-mm disposal biopsy punch (KAI medical) and transferred into 1 mL of Liberase TM (Roche) prepared in basic medium (RPMI + PS + 55 µM BME + 20 mM HEPES), then incubated 2 hours at 37°C. The enzymatic reaction was then stopped with 75 µL of EDTA (100 mM). Cells were homogenized using the Medimachine tissue homogenizer (BD Bioscience) for 8 min and counted using an automated cell counter (BioRad).

### Flow Cytometry Analysis

Cell suspensions were incubated for 15 min at 4°C with Fc Block (BD Biosciences) and then stained for 25 min at 4°C with the following fluorochrome-conjugated antibodies: anti-CD11c-APC-Cy7 (clone: HL3, BD Biosciences) or anti-CD11c-PE (clone REA754, Miltenyi Biotec), anti-MHC-II-VioBlue (clone: M5/114.15.2, Miltenyi Biotec), anti-CD11b-PerCP-Vio700 (clone: REA592, Miltenyi Biotec), anti-EpCAM-PE (clone: caa7-9G8, Miltenyi Biotec) or anti-EpCAM-PE-Vio770 (clone caa7-9G8, Miltenyi Biotec), anti-XCR1-APC-Vio700 (clone REA707, Miltenyi Biotec), anti-PD-L2-PE (clone MIH37, Miltenyi Biotec), CD86-APC (clone PO3.3, Miltenyi Biotec). Dead cells were excluded using Zombie Aqua Fixable Viability Kit (Biolegend). For the analysis of Fc receptor expression, cells were permeabilized using an intracellular fixation & permeabilization kit (eBioscience) and incubated for 25 min at 4°C with anti-CD16(Fc*γ*RIII)/CD32(Fc*γ*RII)-PE-Vio770 (instead of Fc Block, clone: 93, Miltenyi Biotec), anti-FcϵRIα-APC (clone: MAR-1, Miltenyi Biotec), anti-CD23-APC (clone: B3B4, Miltenyi Biotec), anti-CD64-PE-Vio770 (clone: REA286, Miltenyi Biotec). Cells were acquired on a MACSquant 10 or a MACSquant 16 flow cytometer (Miltenyi Biotec) and data were analyzed using FlowJo software using the gating strategies described in **Figures S2** and **S3** (10).

### Confocal Microscopy Analysis

Skin cell suspensions were obtained by Liberase digestion as mentioned above. Cell suspensions were diluted in RPMI and deposited in microplates containing poly-L-lysine-coated coverslips (Corning). Cells were incubated overnight at 4°C and fixed with 4% paraformaldehyde. Cells were then incubated with rat anti-mouse MHC-II (clone 2G9, BD Bioscience) and rabbit anti-clathrin heavy chain (Abcam), followed by AF647 goat anti-rabbit IgG (Invitrogen) and AF-555 goat anti-rat IgG (Invitrogen). Finally, coverslips were mounted on microscope slides using ProLong Gold with DAPI (Life Technologies). Cells were visualized with a LMS 700 confocal microscope (Zeiss) and pictures were edited using Zen software (Zeiss).

### Statistical Data Analysis

Data are presented as median with interquartile ranges. The non-parametric Mann-Whitney test was used to compare unpaired values (GraphPad Prism^®^). Values of p<0.05 were considered significant. The level of significance is indicated with asterisks: *, p<0.05; **, p<0.01; ***, p<0.001; ****, p<0.0001 and n.s., non-significant.

## Results

### Allergen Capture by Skin Dendritic Cells Is Increased in Sensitized Mice, Likely Through the Involvement of Fc Receptors (FcR)

**Figure 1:**
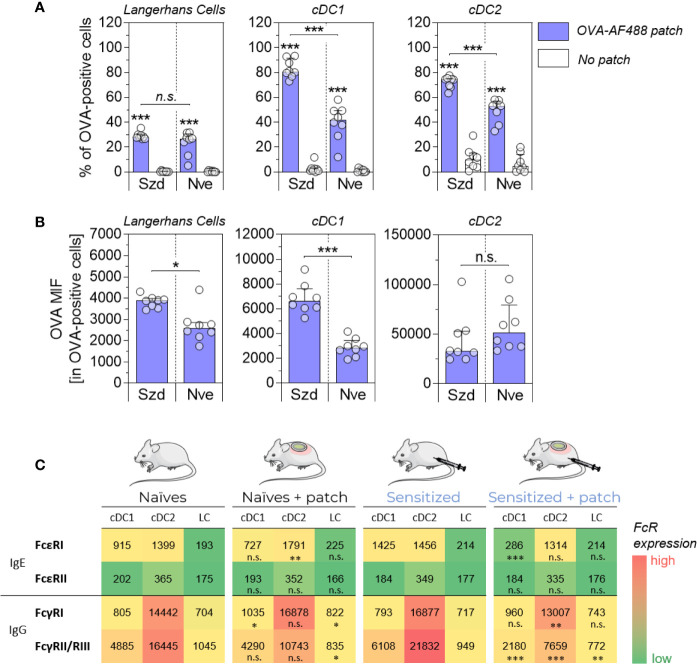
Allergen uptake by skin DC is enhanced in sensitized animals and involves Fc receptors. OVA-sensitized (Szd) or naïve (Nve) mice received a patch containing OVA-AF488 on hair-free skin (depilated) on the back (in blue). As negative controls, mice received no patches (in white). Six hours after patch application, a skin sample corresponding to the patch application area was collected and cells were analyzed by Flow Cytometry. **(A)** The percentage of OVA-positive cells was measured among Langerhans cells, cDC1 and cDC2, as indicated. **(B)** The median of OVA fluorescence intensity (OVA MFI) was measured from OVA-positive DCs. Data are median and interquartile ranges of individual values (N = 8 per group). Data are representative of several independent experiments. **(C)** The relative expression of Fc receptors was evaluated by measuring MFI. Data are median of individual MFI (N = 8 per group). FcϵRI, FcϵRII and FcγRII/RIII expression data are representatives of two independent experiments. The level of significance indicated for sensitized + patch mice was derived from the comparison to sensitized mice. P values were determined using the Mann-Whitney test (*P < 0.05; **P < 0.01; ***P < 0.001; n.s., non-significant).

OVA-sensitized and naïve mice received epicutaneous patches containing fluorescently labeled ovalbumin (OVA-AF488) for 6 hours. Skin cells were then collected and analyzed by flow cytometry (**Figure 1A**). As observed in previous studies, significant increases in the percentage of OVA-positive cDC1 and cDC2 were observed in OVA-sensitized mice compared to naïve animals. There was no significant difference in the percentage of OVA-positive LC observed in OVA-sensitized mice compared to naïve animals. Additionally, a significant increase of the OVA median of fluorescence (MFI) was observed for OVA-positive LC and cDC1 isolated from sensitized mice compared to naïve animals (**Figure 1B**). This suggests that the net amount of OVA antigen captured by these two subsets was greater in sensitized animals. The relative expression of FcR was evaluated by Flow Cytometry in skin DC isolated from naïve mice, naive mice that received an OVA-AF488 patch, OVA-sensitized mice or OVA-sensitized mice that received an OVA-AF488 patch (**Figure 1C**). The analysis was performed on permeabilized cells to allow for the quantification of FcRs that were internalized. In sensitized animals, a significant decrease in the relative expression of FcγRII/RIII (in all DC subsets), FcγRI (in cDC2), and FcϵRI (cDC1) was observed in sensitized mice following patch application. In naïve animals, a slight decrease in the relative expression of FcγRII/RIII was also observed following patch application. However, this decrease was less than that observed in sensitized animals. Note that a similar trend was observed in non-permeabilized cells, although the relative expression of FcϵRI, FcγRI and FcγRII/RIII were slightly lower, likely due to the sole detection of surface receptors (**Figure S4**). Graphs showing individual data points and error bars are available in **Figure S5**. From these results, we hypothesized that FcR, and especially FcγRII/RIII may be involved in the binding of immune complexes formed with OVA and specific antibodies, that then blocked access to immunolabeling antibodies by steric hindrance.

### Specific Humoral Response Is Involved in the Enhancement of Allergen Capture by Skin DC, Leading to an Increase of the Number of OVA-Positive DCs in Local Lymph Nodes

**Figure 2:**
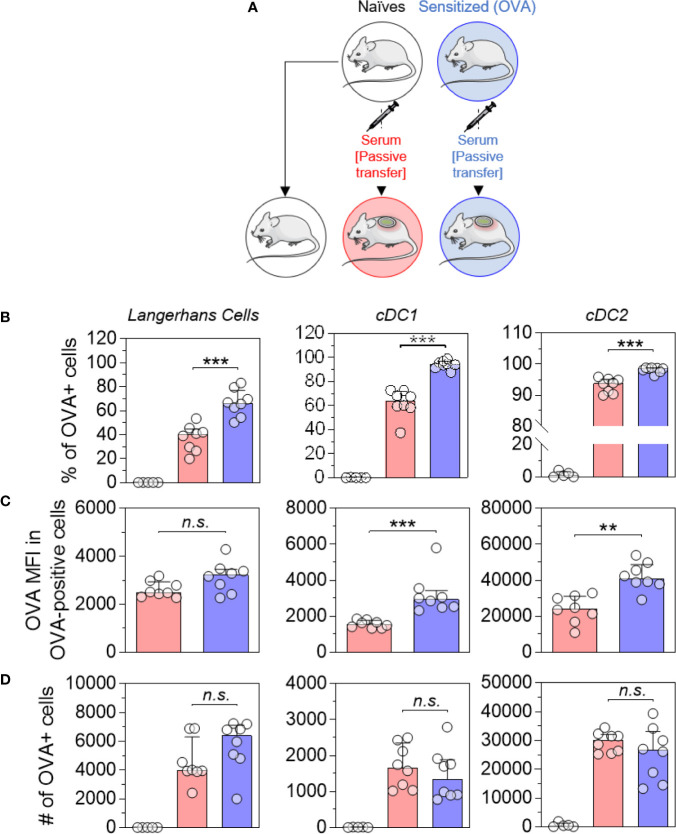
Allergen-specific humoral immunity increases the local uptake of allergen by skin DCs. **(A)** Mice received pooled sera obtained from OVA-sensitized mice (in blue) or naive mice (in red), as indicated. The next day, recipient mice received a patch containing OVA-AF-488 on depilated areas of the back. As a negative control for Flow Cytometric analysis, a group of naïve mice was kept untreated (in white). Six hours after patch application, a skin sample corresponding to the patch application area was collected and cells were analyzed by Flow Cytometry. **(B)** The percentage of OVA positive cells was measured among Langerhans cells, cDC1 and cDC2, as indicated. **(C)** The median of OVA fluorescence intensity (OVA MFI) was measured from OVA-positive cells. **(D)** The absolute number of OVA-positive DC was calculated based on the percentages of OVA-positive cells and the total number of cells in each DC subset. Data are median and interquartile ranges of individual values (N = 8 per experimental group). Data are representative of several independent experiments. P values were determined using the Mann-Whitney test (**P < 0.01; ***P < 0.001; n.s., non-significant).

**Figure 3:**
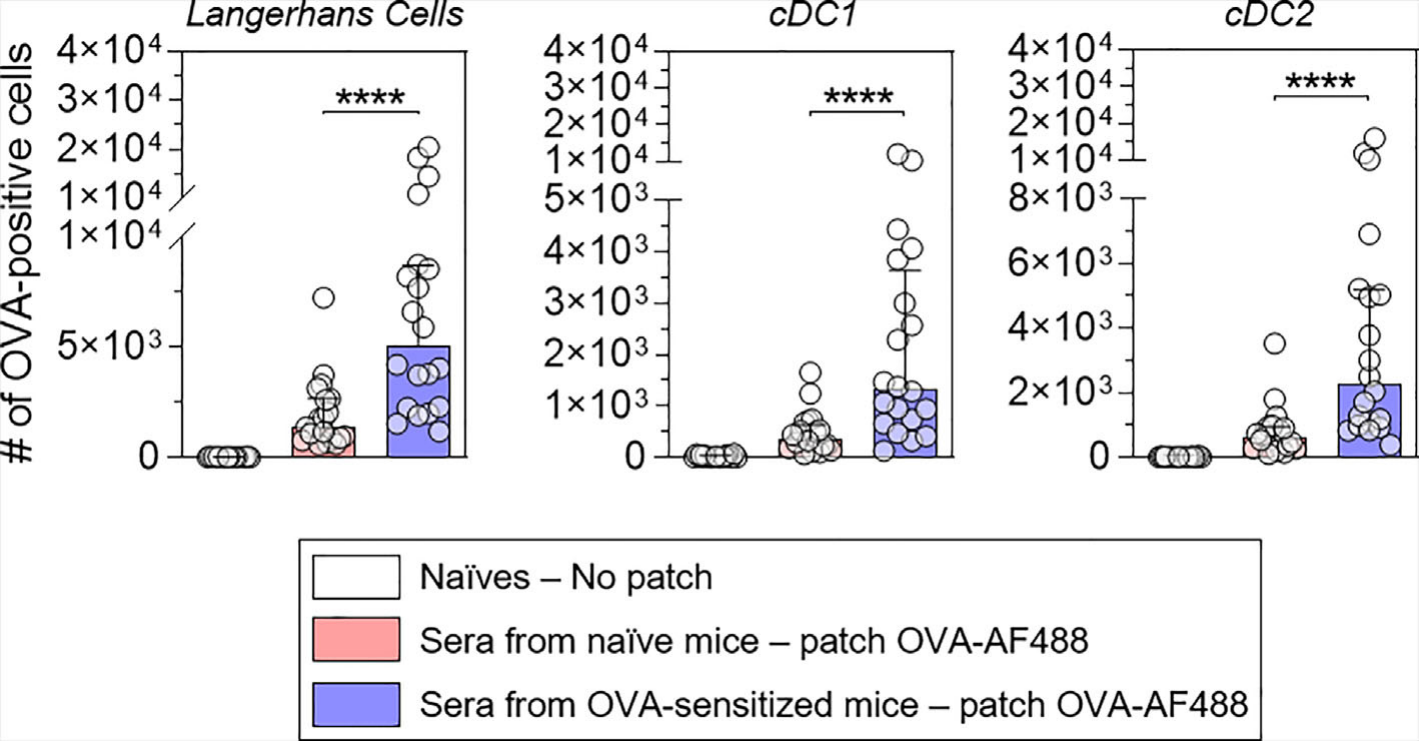
Allergen-specific humoral immunity promotes the increase of the number of OVA-positive migratory DCs in local lymph nodes. Mice were treated as described in **Figure 2**. Forty-eight hours after patch application, brachial draining lymph nodes were collected, and cells were analyzed by Flow Cytometry. The number of OVA positive cells was quantified among migratory Langerhans cells, cDC1 and cDC2, as indicated (pool of two independent experiments, N = 10 per group for each of the two experiments). Data are median and interquartile ranges of individual values. P values were determined using the Mann-Whitney test (****P < 0.0001).

Naïve mice received pooled sera originating from sensitized or naïve mice by intraperitoneal injection (**Figure 2A**). Twenty-four hours after the passive transfer, recipient mice received OVA-AF488 patches. Following 6 hours of patch application, skin cells were collected and analyzed by Flow Cytometry. A significant increase in the percentages of OVA-positive LC, cDC1 and cDC2 were observed in mice that received sera from OVA-sensitized animals compared to mice that received sera from naïve animals (**Figure 2B**). Additionally, an increase of OVA MFI was observed for OVA-positive DCs in mice that received sera from OVA-sensitized animals compared to mice that received sera from naïve animals (significant for cDC1 and cDC2) (**Figure 2C**). Surprisingly, no increase in the absolute number of OVA-positive DCs was found in mice that received sera from OVA-sensitized animals despite the increase in percentages of OVA-positive cells seen (**Figure 2D**). This may reflect a reduction in the total number of DCs in that group, which may result from an earlier migration to local lymph nodes. Following 48 hours of patch application, cells were isolated from BLN and analyzed by flow cytometry (**Figure 3**). A significant increase in the numbers of OVA-positive migratory LC, cDC1 and cDC2 was observed in mice that received sera from OVA-sensitized animals compared to mice that received sera from naïve animals. Overall, these results suggest that the humoral component is the main factor responsible for the increase of allergen capture observed in sensitized animals.

### IgG Is Involved in the Increase of Allergen Capture by Skin DC and Their Migration to Local Lymph Nodes.

**Figure 4:**
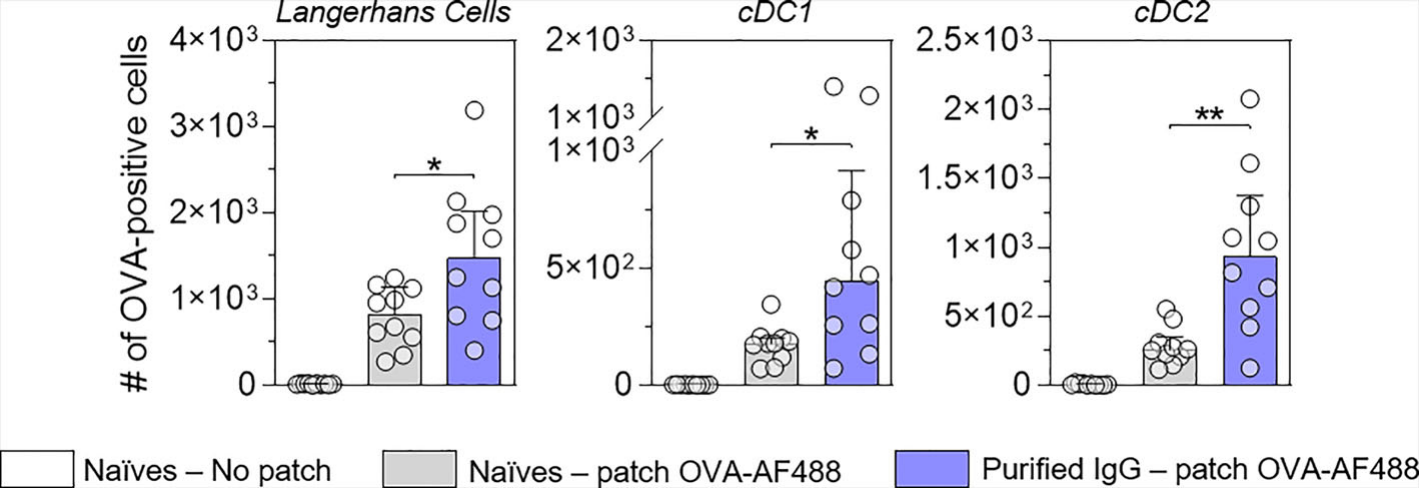
IgG increases the number of allergen-positive DC in local lymph nodes. Mice received purified IgG (in blue) obtained from OVA-sensitized mice. As negative control, mice were kept untreated (in grey). The next day, recipient mice received a patch containing OVA-AF-488 on depilated areas of the back or remained untreated as a negative control (in white). Forty-eight hours after patch application, brachial draining lymph nodes were collected, and cells were analyzed by flow cytometry. The number of OVA positive cells was measured among migratory Langerhans cells, cDC1 and cDC2, as indicated (N = 10 per group, single experiment). Data are median and interquartile ranges of individual values. P values were determined using the Mann-Whitney test (*P < 0.05; **P < 0.01; n.s., non-significant).

**Figure 1** suggests that IgG receptors are mainly involved in allergen capture by skin DC. To assess the role of OVA-specific IgG in the increased number of OVA-positive migratory DC in local lymph nodes, IgG antibodies were purified from a pool of sera originating from OVA-sensitized mice and injected into naïve recipient mice. Twenty-four hours after passive transfer, recipient mice received OVA-AF488 patches (**Figure 4**). Following 48 hours of patch application, cells were isolated from BLN and analyzed by Flow Cytometry. A significant increase in the numbers of OVA-positive migratory LC, cDC1 and cDC2 were observed in mice that received purified IgG compared to untreated mice. Conversely, mice that received IgG-depleted sera originating from OVA-sensitized mice (flow through of IgG purification step containing high amount of IgE) did not show any increase of OVA-positive migratory DCs as compared to mice that received sera originating from naïve mice **(** **Figure S6** **)**. These results suggest that IgG is the main class of immunoglobulin involved in the increase of OVA uptake by skin DC and the greater number of OVA-positive migratory DCs in lymph nodes following OVA-AF488 patch application.

### Blockade of FcγRII/RIII Decreases the Number of OVA-Positive DC at Skin and BLN Levels

**Figure 5:**
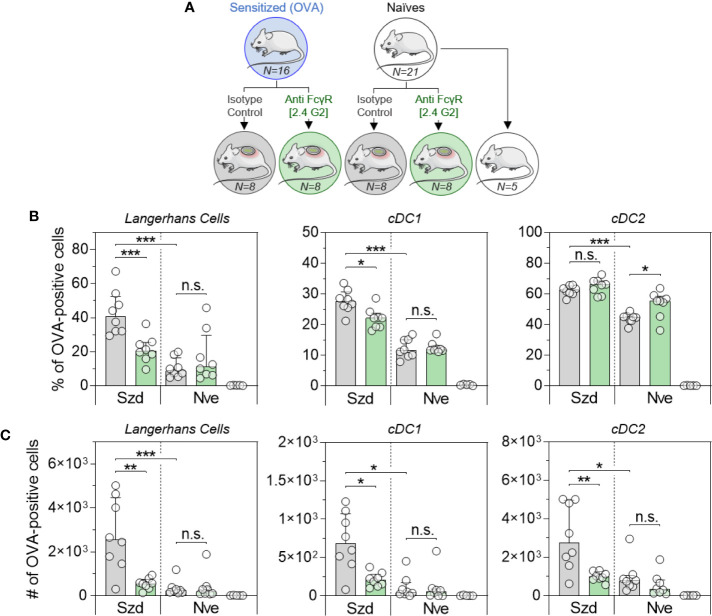
Allergen uptake by skin DC is enhanced in sensitized animals and involves Fc receptors. **(A)** OVA-sensitized (Szd) or naïve (Nve) mice received anti-FcγRII/RIII antibodies (in green) or relevant isotype control (in grey). Twenty-four hours after blocking antibody injection, mice received a patch containing OVA-AF488 on depilated areas of the back (in blue). As a negative control a group of naive mice was kept untreated (in white). **(B)** Six hours after patch application, a skin sample corresponding to the patch application area was collected and cells were analyzed by Flow Cytometry. The percentage of OVA positive cells was measured among Langerhans cells, cDC1 and cDC2, as indicated. **(C)** Forty-eight hours after patch application, brachial draining lymph nodes were collected, and cells were analyzed by Flow Cytometry. The number of OVA positive cells was measured among migratory Langerhans cells, cDC1 and cDC2, as indicated. Data are Median and interquartile ranges of individual values (N = 8 per group, single experiment). P values were determined according to the Mann-Whitney test (*P < 0.05; **P < 0.01; ***P < 0.001; n.s., non-significant).

To further confirm the role of IgG and the involvement of FcγR, OVA-sensitized or naïve mice received anti-FcγRII/RIII blocking antibody or a relevant isotype control. Twenty-four hours after blocking antibody injection, mice received OVA-AF488 patches (**Figure 5A**). Following 6 hours of patch application, skin cells were collected and analyzed by Flow Cytometry (**Figure 5B**). A significant decrease in the percentages of OVA-positive LC and cDC1 were observed in sensitized mice that received blocking antibody compared to mice that received isotype control. Conversely, injection of the blocking antibody did not modify the proportion of OVA-positive LC and cDC1 in naïve animals. Of note, blocking antibody had no or even an inverse effect on cDC2, especially in naïve animals. This suggests that another mechanism may compensate for FcγR blockade in that subset, especially at the skin level. Following 48 hours of patch application, cells were isolated from BLN and analyzed by Flow Cytometry (**Figure 5C**). A significant decrease in the numbers of OVA-positive migratory LC, cDC1 and cDC2 were observed in sensitized mice that received the blocking antibody compared to mice that received an isotype control. Again, the injection of blocking antibodies had no effect in naïve animals. These data suggest that IgG, through the involvement of FcγRII/RIII, is mainly involved in the increase of OVA uptake observed in OVA-sensitized animals following the epicutaneous application of OVA-AF488.

## Discussion

In the present work, we aimed to better understand the role of the preexisting immunologic status in the capture of epicutaneously-administered allergen by skin APC and the subsequent migration of allergen-positive cells to local draining lymph nodes. This study was based on previous observations, showing an increase in the number of OVA-positive DC in the draining lymph nodes of OVA-sensitized mice compared to naïve mice, when OVA is administered epicutaneously (4). Our data strongly suggest that specific antibodies, especially IgG, are mainly involved in this effect through the involvement of FcγR. Of note, although all our data went in the same direction, some of our experiments have been performed once and additional tests would be required to confirm and elaborate on our findings. The increase in the number of OVA-positive migratory DCs observed in the draining lymph nodes of mice that received sera originating from sensitized mice is likely due to an enhancement of migration efficacy but may also be reflective of the higher proportion of OVA-positive DCs in the skin. Previous data demonstrated that the interaction of FcγR with immune complexes can stimulate the migration of DCs from peripheral tissues to draining lymph nodes *via* an increase of CCR7 expression (11). Therefore, in future investigations, it would be worthwhile evaluating whether the passive transfer of sera and/or IgG isolated from sensitized mice would modify the kinetics of skin DC migration to local lymph nodes and modulate the expression of CCR7. In this study, we chose to focus on the three main skin DC subsets (LC, cDC1 and cDC2). However, it may be important to look at other skin APC populations that have been described previously (1, 12, 13).

In previous work, Campana *et al.* demonstrated that epicutaneous allergen application using atopy patch tests was able to boost an allergen-specific cellular response in allergic patients. However, no effect was shown in non-allergic individuals (14). Therefore, the authors suggested that allergen-specific IgE could facilitate allergen uptake by skin DC in humans as it has been previously shown with peripheral blood DC (15). Our data agree with this hypothesis but rather suggest a predominant role for IgG in mice, instead of IgE. This apparent mismatch could be explained by the difference between the two experimental systems that have been used (i.e., Human *versus* mouse). Indeed, our results show that FcϵR are poorly expressed by LC and moderately expressed by dermal DC in mice. This is consistent with previous data suggesting that FcϵR is not expressed in murine LC (16, 17). Overall, our data strongly suggest that IgG antibodies induced by OVA-sensitization form complexes with OVA administered epicutaneously that will be more efficiently captured by FcγR-expressing DC than free OVA. Additional experiments are warranted to confirm and illustrate that assumption. Additionally, it would be interesting to evaluate how these preclinical results are translated to the clinic, especially in patients undergoing EPIT for whom a progressive increase of allergen-specific IgG4 (human equivalent of mouse IgG1) has been observed (18). Our previous data generated in sensitized mice showed that the application of allergen-loaded patches on intact skin leads to an increase of PD-L2 expression and a concomitant decrease of CD86 expression in allergen-positive skin DC (19). Interestingly, this tolerogenic profile is not observed when patches are applied on naïve animals. In line with these observations, our recent preliminary data showed that injection of 2.4G2 blocking antibody prior to patch application had no impact on the subsequent modulation of PD-L2 and CD86 expression in OVA-positive DCs (**Figure S7A**). Of note, in mouse, 2.4G2 blocking antibodies bind to both FcγRIIB, which is an inhibitory receptor, and FcγRIII, which is an activating receptor. Therefore, it would be interesting in future experiments to explore the respective role of FcγRIIB and FcγRIII, as well as the role of other FcRs such as FcγRI, FcγRIV and FcϵRs, in the modulation of skin DC activation between naïve and sensitized individuals. Interestingly, the differential expression of CD86 and PD-L2 observed between sensitized and naïve mice was not seen in OVA-negative DCs, except for CD86 in LC and cDC2 (**Figure S7B**). Of note, the relevance of the results obtained for cDC2 is unclear due to the low proportion of OVA-negative cells measured in that subset. For LCs, however, we cannot exclude an indirect impact of keratinocytes that are known to express several FcγRs (20–23). Future studies should explore the specific role of keratinocytes in LC activation, and how they may be modulated by specific humoral responses. In these future experiments, it would also be relevant to include additional groups of control mice that receive “blank” epicutaneous patches (containing excipient without OVA) to avoid any potential bias linked to patch application and/or excipient (PBS). In addition, it would also be worthwhile addressing whether antigen internalization pathways in skin DCs are different between sensitized and naïve mice and how FcRs may be involved in this internalization. In previous flow cytometry studies performed on permeabilized and non-permeabilized cells, we originally showed that permeabilization leads to a loss of OVA-positive DC subpopulations isolated from naïve mice, while it had no impact on DCs isolated from sensitized animals (**Figure S8**). Therefore, we hypothesized that antigen uptake by skin DCs in sensitized animals followed a pathway involving permeabilization-resistant vesicles. In a preliminary experiment, we evaluated by confocal microscopy whether clathrin vesicles were involved in OVA uptake by skin cells (**Figure S9**). In sensitized animals, we clearly showed intra-cytoplasmic vesicles containing OVA in MHC-II-high cells that might correspond to DCs. By contrast, OVA-positive DCs were very rare in the samples isolated from naïve animals. Although these data confirmed that OVA antigen was internalized by OVA-positive DCs following 6 hours of patch application, we did not show any clear co-localization between clathrin and OVA, suggesting that other pathways are involved.

Our previous data generated in murine and porcine models demonstrated that epicutaneous patches can also be used as a vaccine delivery platform, which is especially efficacious at boosting specific pre-existing immunity (24–26). However, several studies have highlighted the fact that pre-existing immunity, especially antibodies directed against a specific antigen, may interfere with the induction of immune response to an homologous antigen administered as a vaccine (27–31). Interestingly, this interference could be partially overcome by using mucosal routes of immunization such as intranasal administration (32–34). This antibody-mediated interference could result from multiple mechanisms including local antigen destruction by macrophages (35). Our results strongly suggest that pre-existing antibodies did not interfere with the uptake of an epicutaneously-delivered antigen by skin DC, suggesting that the epicutaneous route, similar to the mucosal route of immunization, may alleviate the loss of vaccine efficacy in seropositive individuals. However, in view of these preliminary results, further studies are needed to evaluate the benefit of the epicutaneous route of immunization over the “classical” parenteral route for boosting immune responses by bypassing the interfering effect of preexisting antibodies. If confirmed, this could give a solid advantage to epicutaneous delivery for boosting vaccine responses, and also suggest a role for this delivery route in priming of vaccine responses in seropositive individuals, such as young infants who have maternal antibodies.

## Data Availability Statement

The original contributions presented in the study are included in the article/**Supplementary Material**. Further inquiries can be directed to the corresponding author.

## Ethics Statement

The animal study was reviewed and approved by CEEA127 - DBV Technologies.

## Author Contributions

P-LH, LM, and HS designed experiments. P-LH, CP, NA, NO, LG, AP, AB, VD and J-LL managed and performed experiments. P-LH, LM and HS analyzed data and wrote the manuscript. All authors contributed to the article and approved the submitted version.

## Conflict of Interest

All authors were employed by DBV Technologies at the time of completion of this work. P-LH, CP, J-LL, LM, and HS hold shares in the company.

## Supplementary Material

Supplementary Figure 1 Measurement of IgG1 titers from sensitized donor mice and naïve recipient mice. Blood samples were collected from OVA-sensitized mice and pooled (Szd, donor mice, in blue). This serum pool was injected to naïve recipient mice (Sera Szd, Recipient mice, in blue). As negative controls, mice received pooled sera from naïve mice (Sera Nve, Recipient mice, in red) or were kept untreated (No transfer, in white). OVA-specific IgG1 titers were measured by quantitative ELISA from the pool of sera collected in sensitized donor mice or from blood samples collected in recipient mice, 24 hours after passive transfer. Data are median and interquartile ranges of individual values (N = 6 per group or recipient mice). P values were determined using the Mann-Whitney test (**, P<0.01).

Supplementary Figure 2 Gating strategy used for the analysis of skin cells by FACS. Skin samples were collected 6 hours after patch application and incubated 2 hours at 37°C in 1 mL of Liberase TM prepared in basic medium (RPMI + PS + 55 µM BME + 20 mM HEPES). Then 500 µl of basic medium containing 500 µg/mL of DNase I and 15 mM of EDTA were added to stop the enzymatic reaction and skin samples were homogenized using a Medimachine tissue homogenizer for 8 min. Cells were filtered on 50 µm Filcon and labeled as follow: Cells were incubated 15 min at 4°C with 50 µl of FcBlock in microplates. Cells were washed with MACS buffer and incubated 25 min at 4°C with 50 µl of anti-Epcam-PE-Vio770, anti-CD11b-PerCP-Vio700, anti-MHCII-VioBlue and anti-XCR1-Vio770. Cells were then washed with PBS and incubated 15 min at room temperature with Zombie aqua viability marker. Cells were finally acquired on a MACSquant and gated as described using FlowJo software.

Supplementary Figure 3 Gating strategy used for the analysis of lymph node cells by FACS. The two brachial lymph nodes of each mouse were harvested in 1 mL of FACS buffer in individual petri dishes. One mL of Liberase (0.52U/mL)/DNase I (50µg/mL) in MACS buffer was added in each Petri Dish. Each LN was flushed with a 1 mL syringe, incubated for 20 min at 37°C, and then 250 µl of EDTA 100 mM was added to each Petri Dish to stop the reaction. LN cell suspensions were obtained by dissociation and filtration on a cell strainer (100 µm). Cells were counted, labeled and analyzed as follow: Cells were incubated 15 min at 4°C with 50 µl of FcBlock in microplates. Cells were washed with MACS buffer and incubated 25 min at 4°C with 50 µl of anti-Epcam-PE-Vio770, anti-CD11b-PerCP-Vio700, anti-MHCII-VioBlue, anti-CD11c-PE and anti-XCR1-Vio770. Cells were then washed with PBS and incubated 15 min at room temperature with Zombie aqua viability marker. Cells were finally acquired on a MACSquant and gated as described using FlowJo software.

Supplementary Figure 4 Analysis of Fc receptor expression in non-permeabilized skin DCs. Mice were treated as described in **Figure 1**. The relative expression of Fc receptors was evaluated from non-permeabilized cells by measuring MFI. Data are median of individual MFI (N = 8 per group). The level of significance indicated for patched mice results from the comparison to non-patched mice. P values were determined according to the Mann-Whitney test (*, P<0,05; **, P<0.01; ***, P<0.001; n.s., non-significant).

Supplementary Figure 5 Graphical representation of FcR expression data. Mice were treated as described in **Figure 1**. The relative expression of Fc receptors was evaluated from permeabilized and non-permeabilized cells by measuring MFI, as indicated. Data are median and interquartile range of individual MFI (N = 8 per group). P values were determined according to the Mann-Whitney test (*, P<0,05; **, P<0.01; ***, P<0.001; n.s., non-significant).

Supplementary Figure 6 Passive transfer of IgG-depleted sera does not modify the number of allergen-positive DCs in local lymph nodes. Mice received IgG-depleted sera (in green) originated from OVA-sensitized mice. As negative control, mice received sera originated from naïve mice. The day after, recipient mice received a patch containing OVA-AF488 on depilated back or remained untreated as a negative control (in white). Forty-eight hours after patch application, brachial draining lymph nodes were collected, and cells were analyzed by FACS. The number of OVA positive cells was measured among migratory Langerhans cells, cDC1 and cDC2, as indicated (N = 10 per group). Data are median and interquartile ranges of individual values. P values were determined according to the Mann-Whitney test (n.s., non-significant).

Supplementary Figure 7 Involvement of FcγR has no impact on the tolerogenic profile of skin DC induced by allergen uptake. Mice were treated as described in **Figure 4**. Six hours after patch application, a skin sample corresponding to the patch application area was collected and cells were analyzed by Flow Cytometry. PD-L2 (top panels) and CD86 (bottom panels) expression was evaluated in OVA-positive DCs **(A)** or OVA-negative DCs **(B)** by measuring the median of fluorescence intensity (MFI). PD-L2-PE (clone MIH37, Miltenyi Biotec) and CD86-APC (clone PO3.3, Miltenyi Biotec) were used for cell surface immunolabeling. Data are Median and interquartile ranges of individual values (N = 8 per group, single experiment). P values were determined according to the Mann-Whitney test (*, P<0.05; **, P<0.01; ***, P<0.001; n.s., non-significant).

Supplementary Figure 8 Cell permeabilization leads to the loss of OVA by skin DC isolated from naïve mice. Mice were treated as described in **Figure 1**. Six hours after patch application, a skin sample corresponding to the patch application area was collected and cells were analyzed by flow cytometry. The percentage of OVA-positive Langerhans cells, cDC1 and cDC2 was measured from permeabilized or non-permeabilized cells, as indicated. Data are median and interquartile ranges of individual values (N = 9-10 per experimental group, single experiment). P values were determined according to the Mann-Whitney test (****, P<0.0001).

Supplementary Figure 9 Allergen delivery using epicutaneous patch leads to clathrin-independent allergen uptake by skin DCs. OVA-sensitized or naïve mice received a patch containing OVA-AF488 on depilated back. Six hours after patch application, a skin sample corresponding to the patch application area was collected and homogenized. Cells were deposited on a poly-L-lysine-coated coverslip and labeled with rat anti-mouse MHC-II and rabbit anti-clathrin heavy chain associated to relevant fluorochrome-conjugated secondary antibodies. Cells were acquired on a LMS 700 confocal microscope. A representative photograph of cells isolated from sensitized mice is shown.
