# Supplementary figures and images for: Pre-Existing Humoral Immunity Enhances Epicutaneously-Administered Allergen Capture by Skin DC and Their Migration to Local Lymph Nodes
Source: Front Immunol. 2021 Mar 26;12:609029. doi: 10.3389/fimmu.2021.609029 (PMC8044905; doi:10.3389/fimmu.2021.609029)

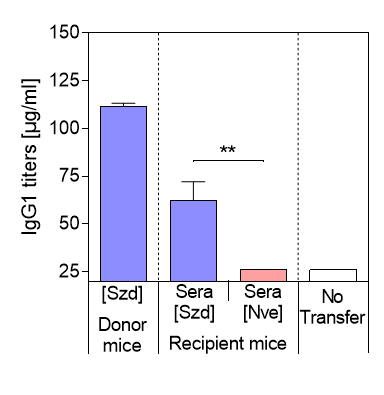

Supplement: Supplementary Figure 1 — Measurement of IgG1 titers from sensitized donor mice and naïve recipient mice. Blood samples were collected from OVA-sensitized mice and pooled (Szd, donor mice, in blue). This serum pool was injected to naïve recipient mice (Sera Szd, Recipient mice, in blue). As negative controls, mice received pooled sera from naïve mice (Sera Nve, Recipient mice, in red) or were kept untreated (No transfer, in white). OVA-specific IgG1 titers were measured by quantitative ELISA from the pool of sera collected in sensitized donor mice or from blood samples collected in recipient mice, 24 hours after passive transfer. Data are median and interquartile ranges of individual values (N = 6 per group or recipient mice). P values were determined using the Mann-Whitney test (**, P<0.01). [file Image_1.tif]

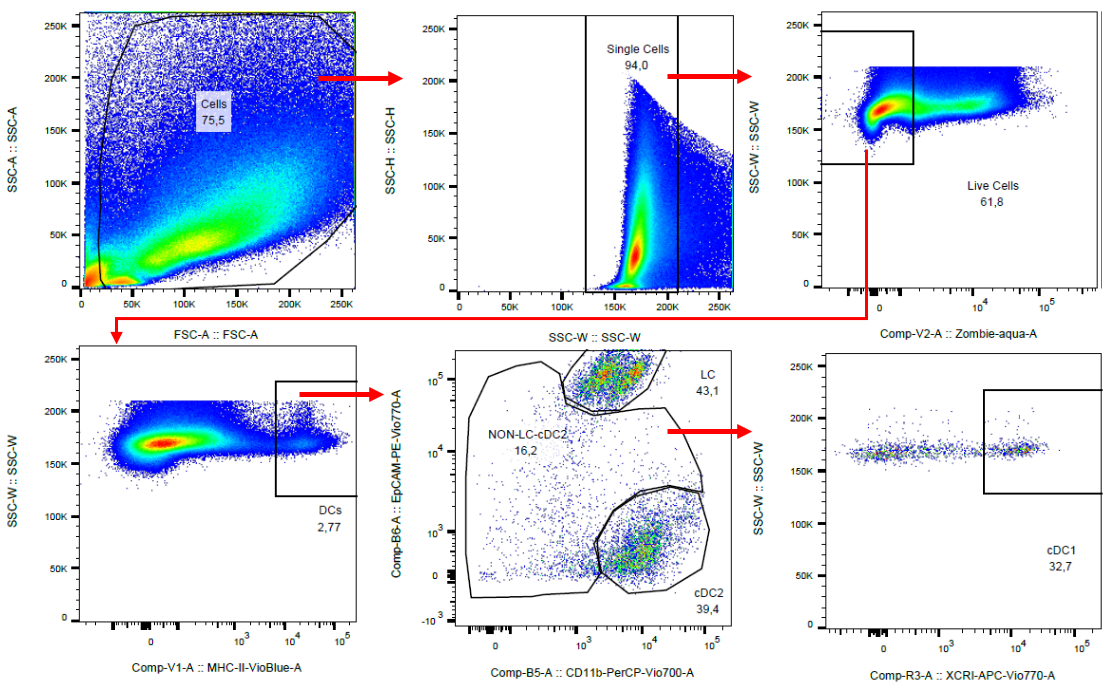

Supplement: Supplementary Figure 2 — Gating strategy used for the analysis of skin cells by FACS. Skin samples were collected 6 hours after patch application and incubated 2 hours at 37°C in 1 mL of Liberase TM prepared in basic medium (RPMI + PS + 55 µM BME + 20 mM HEPES). Then 500 µl of basic medium containing 500 µg/mL of DNase I and 15 mM of EDTA were added to stop the enzymatic reaction and skin samples were homogenized using a Medimachine tissue homogenizer for 8 min. Cells were filtered on 50 µm Filcon and labeled as follow: Cells were incubated 15 min at 4°C with 50 µl of FcBlock in microplates. Cells were washed with MACS buffer and incubated 25 min at 4°C with 50 µl of anti-Epcam-PE-Vio770, anti-CD11b-PerCP-Vio700, anti-MHCII-VioBlue and anti-XCR1-Vio770. Cells were then washed with PBS and incubated 15 min at room temperature with Zombie aqua viability marker. Cells were finally acquired on a MACSquant and gated as described using FlowJo software. [file Image_2.tif]

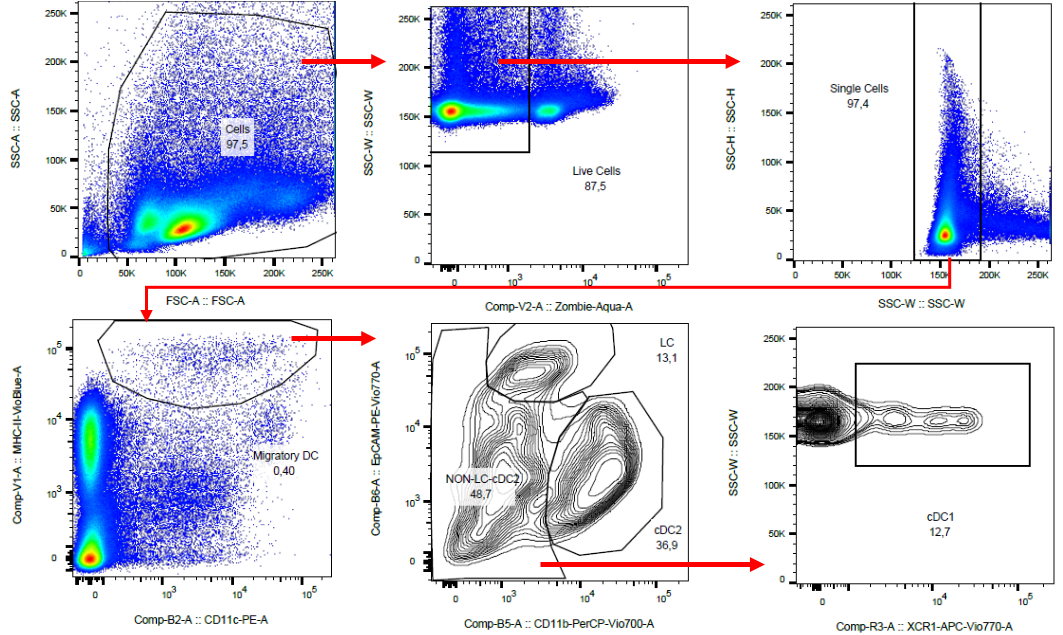

Supplement: Supplementary Figure 3 — Gating strategy used for the analysis of lymph node cells by FACS. The two brachial lymph nodes of each mouse were harvested in 1 mL of FACS buffer in individual petri dishes. One mL of Liberase (0.52U/mL)/DNase I (50µg/mL) in MACS buffer was added in each Petri Dish. Each LN was flushed with a 1 mL syringe, incubated for 20 min at 37°C, and then 250 µl of EDTA 100 mM was added to each Petri Dish to stop the reaction. LN cell suspensions were obtained by dissociation and filtration on a cell strainer (100 µm). Cells were counted, labeled and analyzed as follow: Cells were incubated 15 min at 4°C with 50 µl of FcBlock in microplates. Cells were washed with MACS buffer and incubated 25 min at 4°C with 50 µl of anti-Epcam-PE-Vio770, anti-CD11b-PerCP-Vio700, anti-MHCII-VioBlue, anti-CD11c-PE and anti-XCR1-Vio770. Cells were then washed with PBS and incubated 15 min at room temperature with Zombie aqua viability marker. Cells were finally acquired on a MACSquant and gated as described using FlowJo software. [file Image_3.tif]

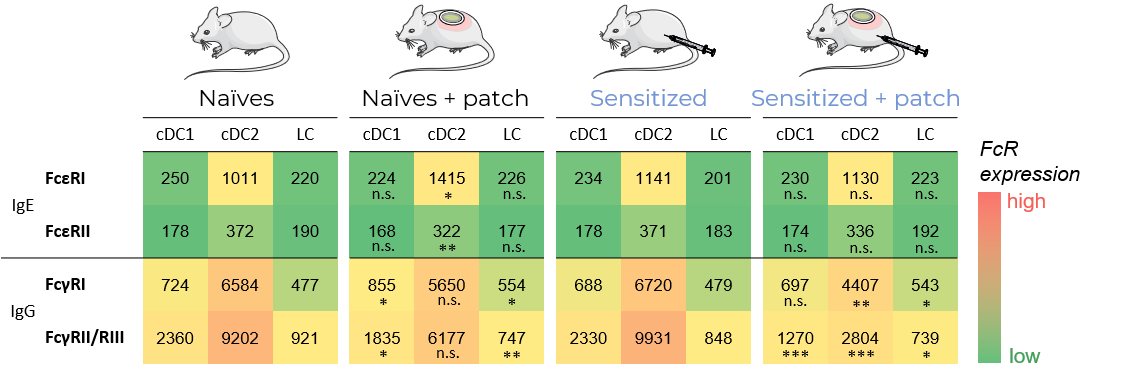

Supplement: Supplementary Figure 4 — Analysis of Fc receptor expression in non-permeabilized skin DCs. Mice were treated as described in Figure 1 . The relative expression of Fc receptors was evaluated from non-permeabilized cells by measuring MFI. Data are median of individual MFI (N = 8 per group). The level of significance indicated for patched mice results from the comparison to non-patched mice. P values were determined according to the Mann-Whitney test (*, P<0,05; **, P<0.01; ***, P<0.001; n.s., non-significant). [file Image_4.tif]

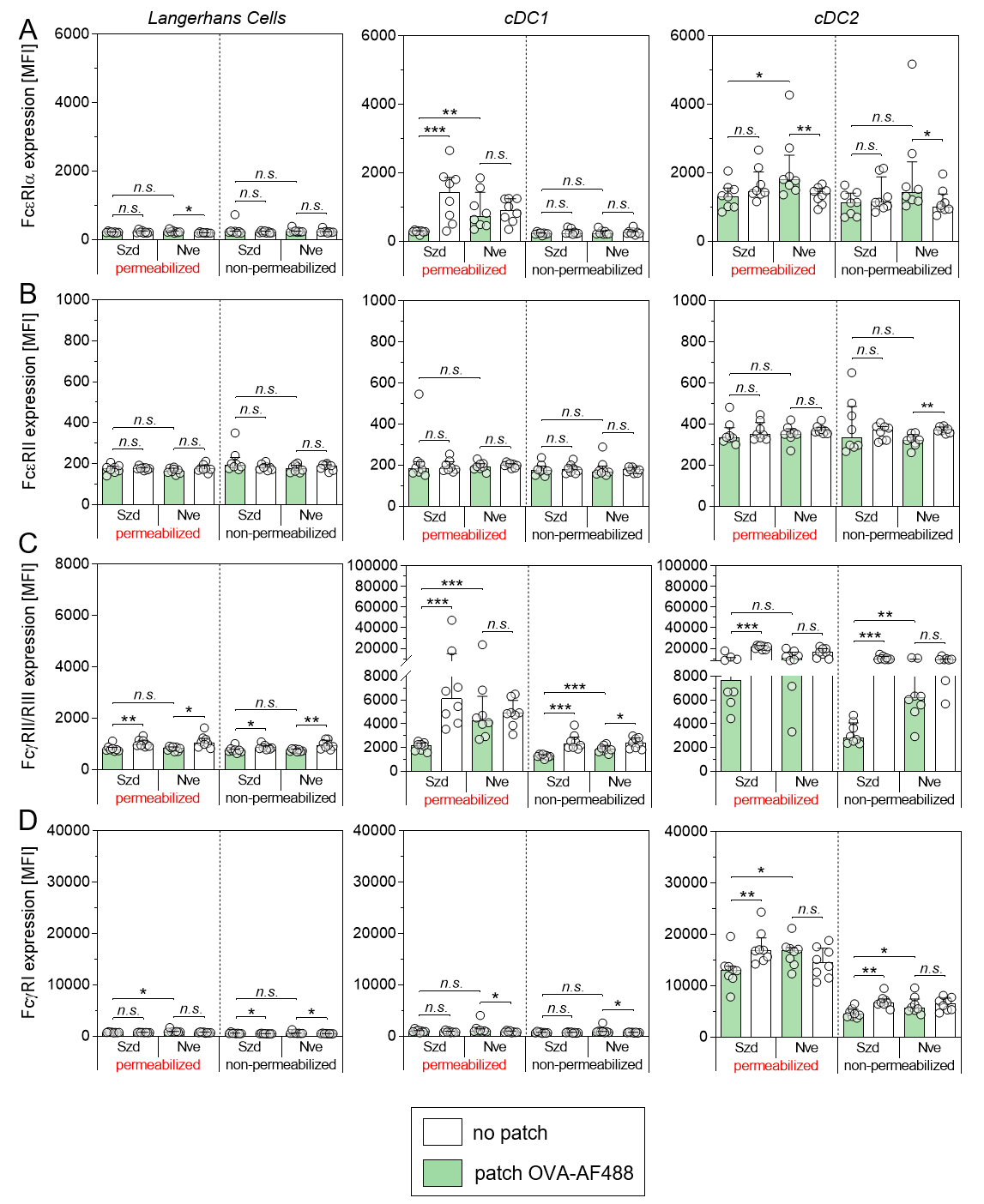

Supplement: Supplementary Figure 5 — Graphical representation of FcR expression data. Mice were treated as described in Figure 1 . The relative expression of Fc receptors was evaluated from permeabilized and non-permeabilized cells by measuring MFI, as indicated. Data are median and interquartile range of individual MFI (N = 8 per group). P values were determined according to the Mann-Whitney test (*, P<0,05; **, P<0.01; ***, P<0.001; n.s., non-significant). [file Image_5.tif]

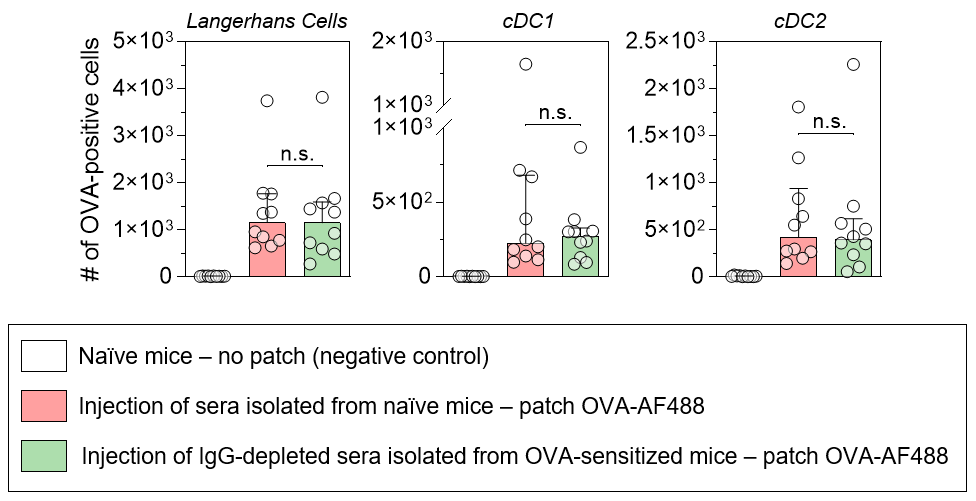

Supplement: Supplementary Figure 6 — Passive transfer of IgG-depleted sera does not modify the number of allergen-positive DCs in local lymph nodes. Mice received IgG-depleted sera (in green) originated from OVA-sensitized mice. As negative control, mice received sera originated from naïve mice. The day after, recipient mice received a patch containing OVA-AF488 on depilated back or remained untreated as a negative control (in white). Forty-eight hours after patch application, brachial draining lymph nodes were collected, and cells were analyzed by FACS. The number of OVA positive cells was measured among migratory Langerhans cells, cDC1 and cDC2, as indicated (N = 10 per group). Data are median and interquartile ranges of individual values. P values were determined according to the Mann-Whitney test (n.s., non-significant). [file Image_6.tif]

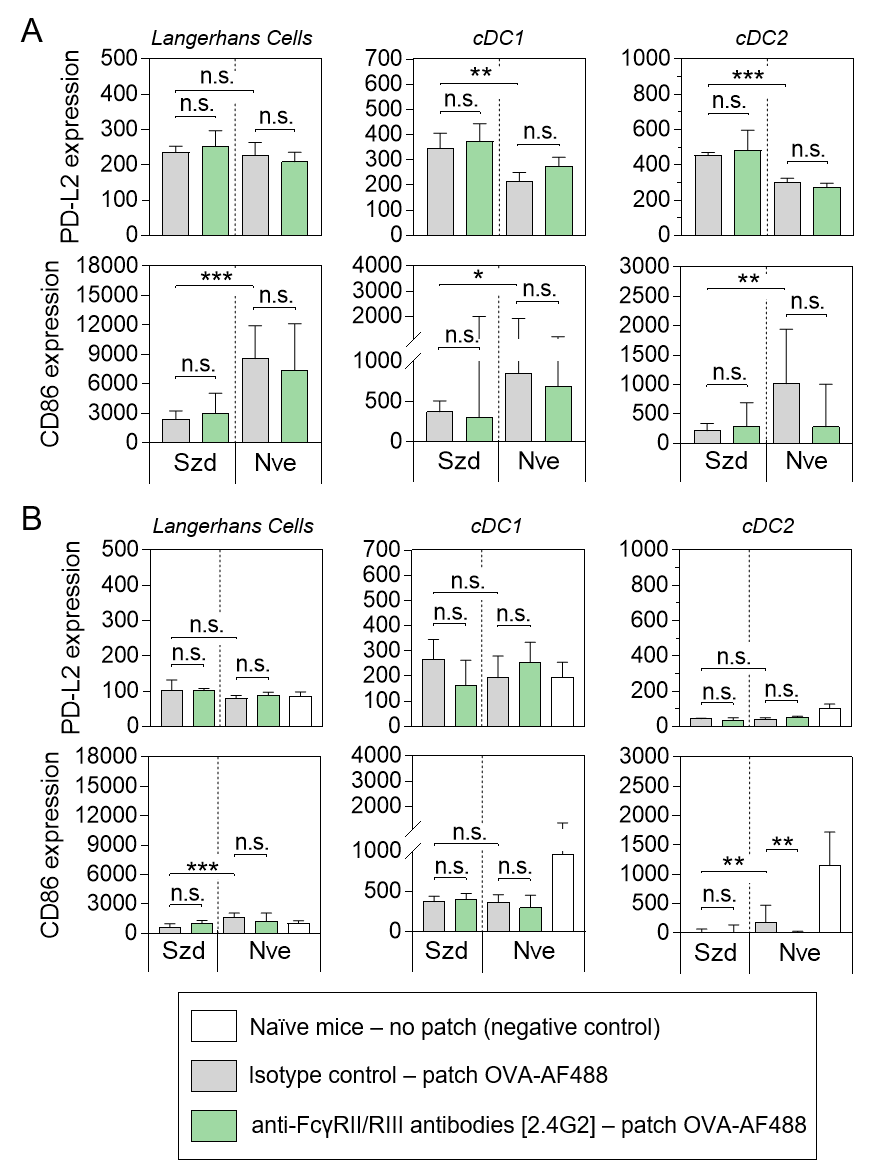

Supplement: Supplementary Figure 7 — Involvement of FcγR has no impact on the tolerogenic profile of skin DC induced by allergen uptake. Mice were treated as described in Figure 4 . Six hours after patch application, a skin sample corresponding to the patch application area was collected and cells were analyzed by Flow Cytometry. PD-L2 (top panels) and CD86 (bottom panels) expression was evaluated in OVA-positive DCs (A) or OVA-negative DCs (B) by measuring the median of fluorescence intensity (MFI). PD-L2-PE (clone MIH37, Miltenyi Biotec) and CD86-APC (clone PO3.3, Miltenyi Biotec) were used for cell surface immunolabeling. Data are Median and interquartile ranges of individual values (N = 8 per group, single experiment). P values were determined according to the Mann-Whitney test (*, P<0.05; **, P<0.01; ***, P<0.001; n.s., non-significant). [file Image_7.tif]

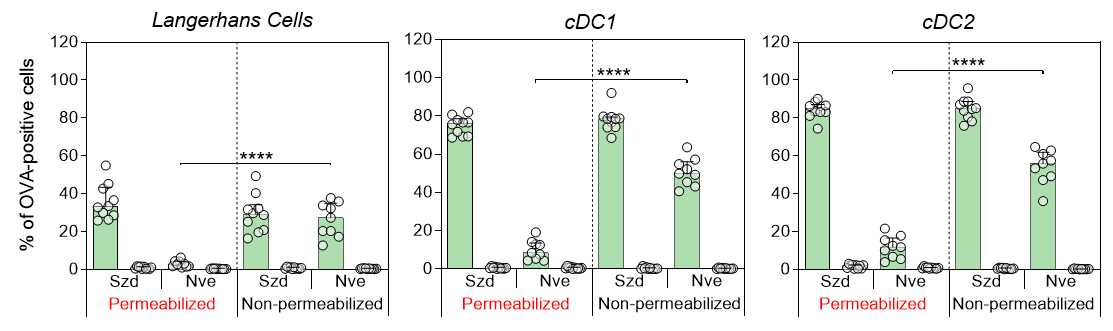

Supplement: Supplementary Figure 8 — Cell permeabilization leads to the loss of OVA by skin DC isolated from naïve mice. Mice were treated as described in Figure 1 . Six hours after patch application, a skin sample corresponding to the patch application area was collected and cells were analyzed by flow cytometry. The percentage of OVA-positive Langerhans cells, cDC1 and cDC2 was measured from permeabilized or non-permeabilized cells, as indicated. Data are median and interquartile ranges of individual values (N = 9-10 per experimental group, single experiment). P values were determined according to the Mann-Whitney test (****, P<0.0001). [file Image_8.tif]

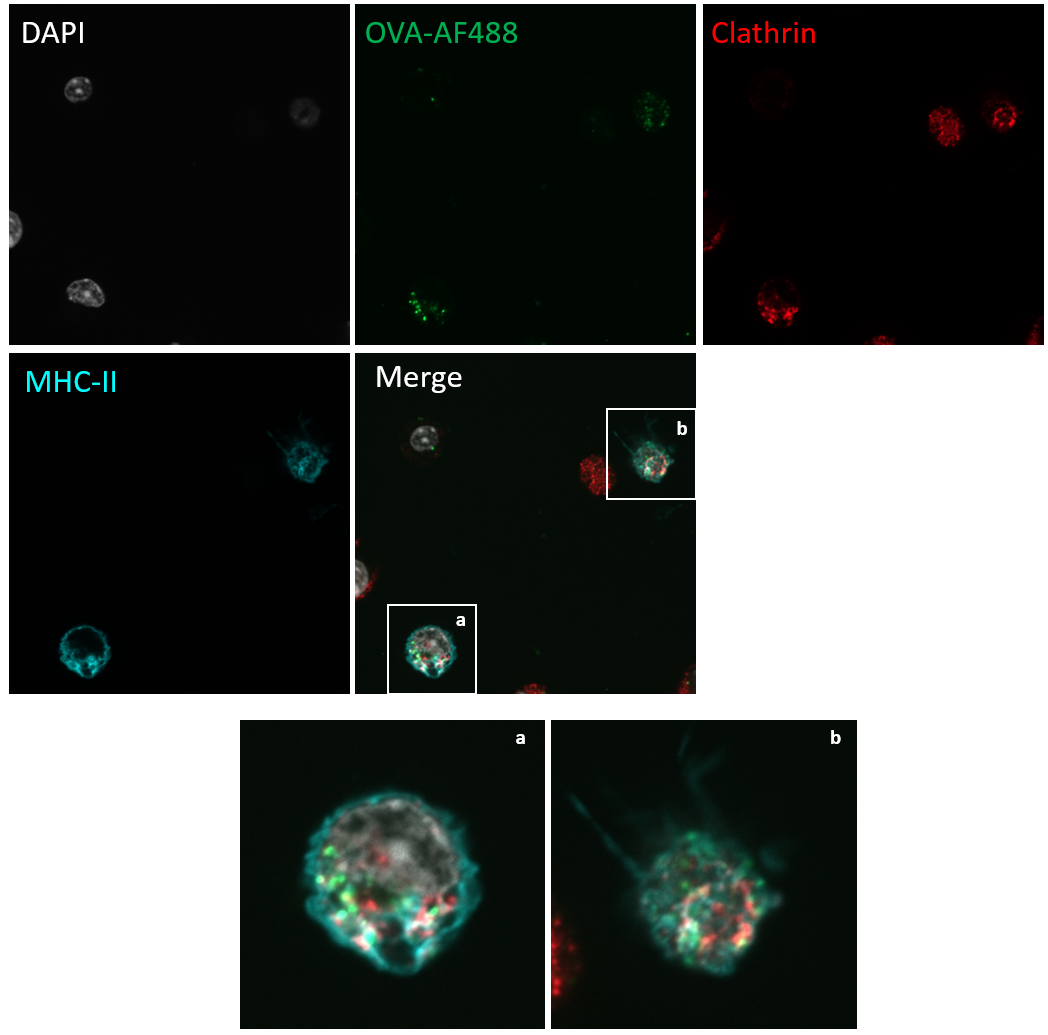

Supplement: Supplementary Figure 9 — Allergen delivery using epicutaneous patch leads to clathrin-independent allergen uptake by skin DCs. OVA-sensitized or naïve mice received a patch containing OVA-AF488 on depilated back. Six hours after patch application, a skin sample corresponding to the patch application area was collected and homogenized. Cells were deposited on a poly-L-lysine-coated coverslip and labeled with rat anti-mouse MHC-II and rabbit anti-clathrin heavy chain associated to relevant fluorochrome-conjugated secondary antibodies. Cells were acquired on a LMS 700 confocal microscope. A representative photograph of cells isolated from sensitized mice is shown. [file Image_9.tif]
